# Functional outcomes characterising mild, moderate and severe hallux valgus

**DOI:** 10.1186/1757-1146-6-S1-O30

**Published:** 2013-05-31

**Authors:** Sheree Nix, Bill Vicenzino, Michelle Smith

**Affiliations:** 1 School of Health and Rehabilitation Sciences, The University of Queensland, Brisbane, QLD, 4072, Australia; 2 School of Clinical Sciences, Queensland University of Technology, Brisbane, QLD, 4059, Australia

## Background

Previous studies investigating functional performance and plantar pressures in HV have reported inconsistent findings. This study investigated functional performance, muscle strength and plantar pressures in otherwise healthy adults with mild, moderate and severe HV compared to controls.

## Methods

Sixty adults with HV and 30 controls participated. Functional measures included: hallux muscle strength, walking performance, postural sway and forefoot plantar pressures. Cluster analysis was used to classify HV subjects as mild, moderate or severe based on radiographic HV angle. Multiple analysis of covariance and pairwise comparisons (*P*<0.05, Bonferroni adjustment) were used to investigate differences between groups, adjusting for age, gender, body mass index and foot pain.

## Results

In those with moderate and severe HV, we found reduced hallux plantarflexion (mean differences (MDs) -50.1N to -45.8N) and abduction strength (MDs -12.3N to -11.2N) compared to controls (*P*≤0.01). A significant reduction in hallux peak pressure (PP) and pressure-time integral (PTI) was evident in moderate HV (MD: PP -90.8kPa; PTI -18.3kPa*s) and severe HV (MD: PP -106.2kPa, PTI -24.4kPa*s) compared to controls (*P*<0.01). Those with severe HV demonstrated increased mediolateral postural sway in single leg stance compared to controls (MD 3.5cm, *P*=0.01). There were no significant differences in walking performance across groups (*P*>0.05).

## Conclusion

Adults with moderate to severe HV may have reduced hallux plantar pressures and muscle strength, whereas those with mild HV appear to function similarly to controls on these parameters. It is important to consider severity of deformity in HV, and target interventions towards specific functional deficits.

